# Degrees and Diplomas

**Published:** 1909-04-17

**Authors:** 


### Editor's Letter-Box.

### DEGREES AND DIPLOMAS.

*To the Editor of* The Hospital.

Sir,—As a medical man who is neither a graduate of London University nor a diplomate of the Royal Colleges, I should like to offer a few comments from an outsider's position upon the relations between these two corporations as discussed in your leading article of April 10.

The M.D. degree of London University is beyond doubt more highly esteemed of the English laity than any other medical degree in the world. This esteem has been obtained by setting, ever since the foundation of the University, a standard which is admittedly very high, though at the present time it is held by many authorities that the Oxford and Cambridge degrees both academically and practically are dangerous rivals. It is, we all admit, a fact that no amount of book learning and no degree can of themselves make a successful clinician; and that some men who have no degree are deservedly more successful than some men who have. That is no argument against the maintenance of a high level of examination proficiency for the M.D.; and it is no argument in favour of equalising the alphabetical suffixes of those who have and those who have not obtained this coveted distinction. It must be recognised that to grant the London M.D. to those who can pass the present standard for the colleges will certainly destroy the commercial value of the degree. The public cannot distinguish between Pass degrees and Honours degrees: what they look at is the superscription of the brass plate, the endorsement of a cheque, or the signature of a certificate, and there both classes would appear simply as M.D.

The trouble is, as we all know, that at many provincial universities degrees are awarded to candidates whose proficiency, as tested by examinations and otherwise, is not only no greater than that of the College diplomates, but is often distinctly inferior. In other words, these universities are letting their degrees go much too cheap. This policy has for some years been attracting students who would otherwise have come to London; but it is, besides a real disservice to medical education, of very doubtful expediency in the long run. It has resulted in a distrust of the degrees in question, and an enhancement of the public confidence in those of London University. Meanwhile, it is, as you say, esteemed a "decided hardship" by students in London that they obtain only diplomas for an expenditure of money, time, and energy which elsewhere will procure them a degree. But it must be emphasised that they can all, if they but will, obtain the London degree; and that its extra value has been created by the extra work necessary to secure it. By asking for the M.D. degree after a course of study no more arduous than that which now secures for them the M.R.C.S., L.R.C.P., they are asking the London graduates to share with them a definite commercial advantage which the latter, as a body, have made for themselves by the toil of their brains, if not by the sweat of their brows. Further, if they obtain the desired prize on these terms they not only prejudice the property of the present graduates, but they will not even long enjoy any advantage themselves; for the public will soon cease to attach any particular value to this degree. The success in recent years of the Cambridge school has not been built up by such means: it has been the steady rise of standard there which has attracted more and more students and made them eager to possess the medical degrees of that University.

In making these remarks I am open to the reply that I offer no alternative scheme, and that something must be done if London schools are not to be emptied altogether. That criticism I must admit; but what, in my opinion, ought to be done, and eventually will be done, is the unification of all examinations for medical qualification throughout the kingdom, and indeed throughout the Empire. To allow a score or so of competing corporations to tout for students and revenue by cutting down the standard of their medical degrees to the lowest limit which the inspectors of the General Medical Council will tolerate is a spectacle which is to me revolting. I fully agree with your remarks about the waste of money and energy due to the separate examinations of the University and the Colleges in London; but on the same analogy it can be argued that Licentiates of the Apothecaries' Hall should be allowed the London M.D. If the London University degrees must be watered to save the London schools, at least the process might be confined to the M.B. The honours course in this would then still be a necessary preliminary to the doctorate, and so the latter might be saved from the catastrophe, as I regard it, with which it is threatened.

Apologising for the length of this letter, and enclosing my card, I shall ask you to preserve me from the wrath of diplomates under the anonymity of

Yours faithfully,

London, April 8, 1909. North Country.

THE HOSPITAL April 17, 1909.

